# A computer vision approach for the grading of cotton base load ages in measuring the performance of washing machine

**DOI:** 10.1371/journal.pone.0342045

**Published:** 2026-04-10

**Authors:** Shaojin Ma, Xue Bai, Yan Bai, Jiajia Shao

**Affiliations:** 1 China National Institute of Standardization, Beijing, China; 2 Key Laboratory of Energy Efficiency, Water Efficiency and Greenization, State Administration for Market Regulation, Beijing, China; The University of Texas, MD Anderson Cancer Center, UNITED STATES OF AMERICA

## Abstract

The performance testing standards for washing machines specify clear requirements regarding the age of the base load used. To enable non-contact detection of the service life of the test fabric and thereby improve the consistency of washing machine performance test results, this study employed computer vision techniques to investigate the feasibility of using image features of the base load for age grading. Base load samples underwent 1–100 accelerated washing cycles were categorized into five degradation stages (C1–C5 were used to represent base loads with ages of 1–20 cycles, 21–40 cycles, 41–60 cycles, 61–80 cycles, and 81–100 cycles, respectively). Wrinkle information and plain weave structure information were extracted from base load images, from which color, texture and area features were obtained. In addition, k-nearest neighbors (kNN), multilayer perceptron (MLP), linear discriminant analysis (LDA), and logistic regression (LR) classifiers were trained to grade the age of base load. As a result, LR classifier demonstrated robust overall performance, achieving accuracy of 0.75, 0.75, 0.62, 0.75, and 1.00 for C1, C2, C3, C4, and C5, respectively. Models utilizing exclusively plain weave structure-derived features consistently outperformed those using only wrinkle-derived features across all classifiers. These results validate computer vision as an effective tool for objective base load aging assessment, offering significant potential to streamline washing machine testing protocols and enhance sustainability. Future work can be focused on expanding sample sizes and exploring mobile-based implementation.

## Introduction

For centuries, laundering clothes required labor-intensive manual efforts, demanding hours of scrubbing, rinsing, and wringing. Washing machines have become a ubiquitous household device, offering an efficient and time-saving alternative to traditional hand washing. Different types of washing machines, such as impeller type, drum type, and mixer type, have varying washing performance [1]. To assist consumers in their selection and for market regulation in China, each washing machine must undergo performance testing before being released to the market. The performance testing methods for electric washing machines in Chinese National Standards (GB/T 4288−2018) are similar to the International Electrotechnical Commission (IEC) standards. According to the standard published in IEC (IEC 60456), the performance of the washing machine includes standard cleaning performance, remaining moisture content, rinsing performance, energy consumption, water consumption, program duration, etc. The washing performance test is conducted by operating the washing machine with standard textile test materials and the detergents under certain conditions. Standard test materials include base loads and standard stain strips, which were attached to the base loads to simulate contaminated clothing.

According to GB/T 4288−2018, the base load consists of a specified quantity of sheets, shirts, towels, and handkerchiefs, all of which are cotton products with identical yarn count and fabric weight. It has been reported that degradation of fabric would occur during washing, which is caused by the abrasion of wet fabric and cleaning agents [2]. The textile type, textile weight, detergent dosage can affect the microfiber emissions from washing machines [3]. To minimize the influence of changes in the characteristics of the base load items with increasing age, the recent standard published in IEC has required the average age for cotton base load items. For each test run, load items should be well distributed in age for each different item type to give a weighted average age of the base load between 20 test runs and 60 test runs. To achieve the weighted average age requirements for a cotton base load, base load items are composed of a mixed age load for: a) 1–20 cycles, 21–40 cycles, 41–60 cycles, and 61–80 cycles. However, it was found that rotation of testing personnel or simultaneous evaluation of multiple washing machines significantly complicates the management of base load age. The IEC standard recommends the use of electronic systems, such as RFID technology, to track the number of tests for each base load. However, such a physical-contact-based testing approach is highly likely to interfere with the washing process during testing. A low cost and rapid base load age measurement method is crucial to effective management of energy labeling.

Computer vision techniques have attracted increasing attention in the field of environmental science [4,5], biology [6], precision agriculture [7], etc. Computer vision systems can replace human visual inspection by using cameras to capture images combined with image processing algorithms, delivering more objective inspection results compared to manual assessments. In the field of textile inspection, computer vision has also been extensively utilized [8]. For instance, an image analysis system for automatic grading of color fabric wrinkling was proposed by [9]. Six image features were extracted and used to linear regression model. The relationship between wrinkle degree and the six features was established, with a coefficient of correlation of 0.982. Recently, a multi view imaging-based method has been proposed to evaluate fabric wrinkle [8]. The improved patch-based multi-view stereo (PMVS) algorithm was used to reconstruct the multi-view fabric surface image sequence. A two-dimensional depth image was generated with four texture feature parameters being extracted, which were then used to develop a support vector machine model (SVM) for the classification of wrinkle grade. The total recognition accuracy of SVM for fabrics categorized into five wrinkle grades can reach 89.86%. The above research demonstrates that appropriate image information can be used to assess the wrinkling degree of fabrics. However, current approaches to establishing grading models for fabric wrinkles all rely on correlating extracted image features of various fabrics with human-assigned scores (visual assessment). The changes in image characteristics of cotton loads as their age in washing machines increases remain unclear. Furthermore, these studies have primarily focused on extracting the skeleton or profile of wrinkles on the fabric surface, without exploring the potential contribution of other types of information to model development.

To support the efficient management of test fabrics in standard performance tests for washing machines, this work explored the feasibility of using computer vision technique to evaluate the age of base load employed in washing machine performance standard testing. Base load samples were prepared according to the following washing machine cycles: 1–20 cycles, 21–40 cycles, 41–60 cycles, 61–80 cycles, and 81–100 cycles. Wrinkles and plain weave structures information of the base load image were extracted for developing machine learning models. Image features derived from wrinkles and plain weave structures information were used as input of four types of machine learning classifiers. Finally, performance of different classifiers and image information were compared and discussed.

## Materials and methods

### Washing machine and base load

For the performance testing of washing machine, a SIEMENS (WJ45XMY88W) instrument was employed, with a maximum load capacity of 10 kg. In this experiment, the base loads were operated under half-load conditions, i.e., configured as 5 kg of 100% cotton bed sheet, towel, shirt, and handkerchief. In specific, 2 bed sheets, 4 shirts, and 31 towels, along with 10 towels and 5 handkerchiefs serving as load counterweights, were prepared. All load items were folded following standardized procedures specified in GB/T 4288−2018, which references the standard published in IEC. In detail, the bed sheets were folded into thirds to form a letter “Z” by the following steps: 1) grasp the bed sheet in the center, 2) shake the bed sheet so that it hangs loosely, 3) fold it twice to a third of its total size, 4) and lightly compress the folded bed sheet before placing it into the drum. The shirts were folded in the following steps: 1) grasp the shirt in the center, and 2) shake the shirt so that it hangs loosely. The towels and handkerchiefs were folded following the procedure analogous to that of the shirts. Standard stain strip was not employed in this study.

The base loads were loaded according to the loading requirements defined in GB/T 4288−2018. The sequence and number of items for each step is given in Table 1. The bed sheets were placed as a lying “Z” perpendicular to the washing machine axis. Two shirts were laid alternately in opposite directions on the same level. The towels and handkerchiefs were placed into the drum from back to front parallel to the drum axis. If several items were required, they were placed with alternating orientation, as can be seen in S1 Fig. Each layer of the base load configuration was manually pressed downward with gentle force following placement.

**Table 1:** Loading procedure of the base loads.

Step	Base load item	Number of items
1	Bed sheet	1
2	Towel	5
3	Shirt	2
4	Towel	10
5	Towel	5
6	Bed sheet	1
7	Towel	5
8	Shirt	2
9	Towel	10
10	Towel	6
11	Handkerchiefs	5

Standard powder detergent was used in this experiment. The detergent was added in the washing machine for each single test run. Following detergent addition, the washing machine was initiated in the Cotton 60℃ programme, with a complete cycle duration of 1 h. The test conditions were controlled according to IEC standards.

### Image acquisition

According to IEC 60456:2024, a weighted average age of the load between 20 and 60 test runs should be attained. The test loads include those subjected to 1–20 cycles, 21–40 cycles, 41–60 cycles, 61–80 cycles. Therefore, the ages of base loads were classified into 5 grades according to the running cycles of the washing machine, namely: 1–20 cycles, 21–40 cycles, 41–60 cycles, 61–80 cycles, and 81–100 cycles (Denoted as C1, C2, C3, C4, C5, respectively). Images of the base load were acquired over a 0-to-100-hour testing period. Initially, a brand-new base load was imaged (i.e., at 0 h). Thereafter, starting from 5 h of operation, image data were collected every 5 hours (i.e., at 5 h, 10 h, …, up to 100 h). It should be noted that, after every 5-hour washing cycle, the base loads were smoothly hung to air-dry to avoid wrinkles caused by factors other than the washing process. During each image acquisition session, 10 dried towels were captured, thus each grade comprised 40 images (4 sampling intervals × 10 images). In total, 200 images (5 grades × 40 images/grade) were collected. Each image had a resolution of 1280 × 1024 pixels.

**Fig 1:**
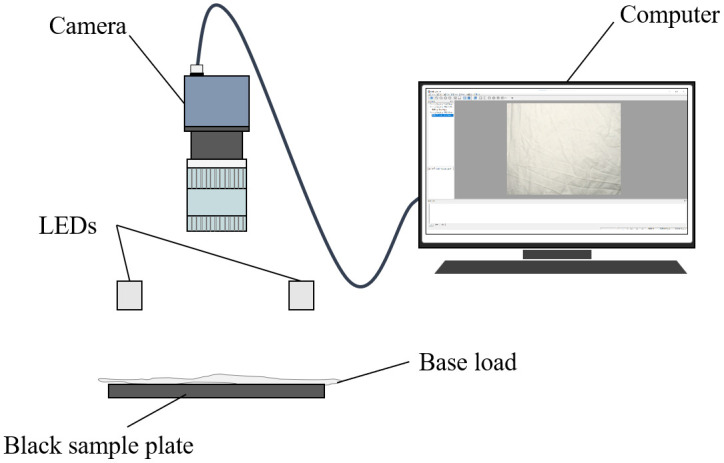
Diagram of the image acquisition setup.

Images of the cotton base loads were acquired using a self-developed computer vision system, which consisted of a camera (MER2–135-208U3C, Daheng Imaging, China) mounted with an 8 mm focal lens, two strip white LEDs, a black sample plate, and a computer. Fig 1 shows the diagram of the image acquisition setup. To avoid interference from the color of the black sample plate on imaging, towel samples were selected for image analysis. Each towel sample was folded twice before imaging, and logos on the base loads were excluded. The camera was placed 30.0 cm above the sample plate. The two strip LEDs were symmetrically arranged along the optical axis of the camera lens. The operating voltage and current of the two LEDs were 21.00 V and 0.13 A, respectively. The exposure time of the camera was 10000μs. Automatic white balance calibration of the camera was performed prior to all image acquisition procedures, obtaining white balance coefficients of 2.0625, 1.0000, and 1.6367 for the R, G, and B channels, respectively.

### Image feature extraction

**Fig 2:**
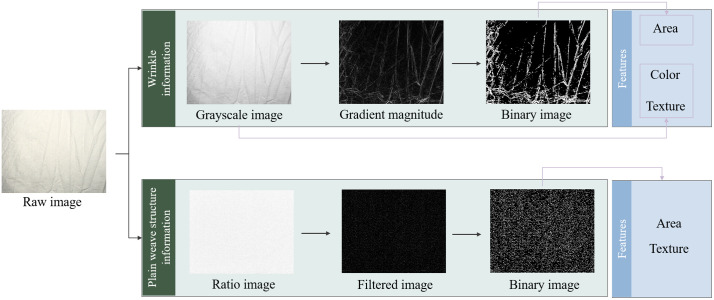
Flow chart of base load image feature extraction.

Image features were extracted from the information of wrinkles and plain weave structures of the base load. Fig 2 shows the flow chart of the image processing step. When extracting image information of wrinkles, the plain weave structure information was suppressed, and vice versa. For wrinkles information extraction, the raw image was firstly transformed to a grayscale image. Contrast-limited adaptive histogram equalization (CLAHE) was applied to the grayscale image, followed by Gaussian smoothing filters. The gradient magnitude of the filtered image was then computed and used to attain a binary image. The binary image can characterize the skeleton of the wrinkles of base load. In addition, gray-level co-occurrence matrix (GLCM) was created from the binary image for texture feature extraction. The offsets were specified as [0 1; −1 1; −1 0; −1–1]. Therefore, four GLCMs were created from each grayscale image. Finally, 6 types of image features (namely, the LBP and area features used to characterize plain weave structure; the entropy, area, and GLCM features used to characterize wrinkles, as well as statistical features.) were extracted. Prior to classifier input, three features with value of 0 were deleted, which belong to the LBP features. Consequently, 30 image features were finally obtained and used to train the grading model, as summarized in Table 2.

**Table 2:** Image features extracted from base load image.

Information	Type	Feature number	Name
Plain weave structure	Texture	1	LBP feature
		2	LBP feature
		3	LBP feature
		4	LBP feature
		5	LBP feature
		6	LBP feature
		7	LBP feature
	Area	8	Plain weave area
Wrinkles	Texture	9	Entropy
	Area	10	Total wrinkle skeleton area
	Texture	11	Contrast of GLCM using offset of [0 1]
		12	Contrast of GLCM using offset of [−1 1]
		13	Contrast of GLCM using offset of [−1 0]
		14	Contrast of GLCM using offset of [−1–1]
		15	Correlation of GLCM using offset of [0 1]
		16	Correlation of GLCM using offset of [−1 1]
		17	Correlation of GLCM using offset of [−1 0]
		18	Correlation of GLCM using offset of [−1–1]
		19	Energy of GLCM using offset of [0 1]
		20	Energy of GLCM using offset of [−1 1]
		21	Energy of GLCM using offset of [−1 0]
		22	Energy of GLCM using offset of [−1–1]
		23	Homogeneity of GLCM using offset of [0 1]
		24	Homogeneity of GLCM using offset of [−1 1]
		25	Homogeneity of GLCM using offset of [−1 0]
		26	Homogeneity of GLCM using offset of [−1–1]
	Statistical features	27	Mean color value of grayscale image
		28	Standard deviation color value of grayscale image
		29	Skewness of grayscale image
		30	Kurtosis of grayscale image

For plain weave structure information extraction, a ratio image calculated using B/G channel was first obtained to weaken the information of wrinkles. Then, a filtered image was created using the raw and transposed Sobel filter, which emphasizes horizontal (Sobel filter) and vertical edges (transposed Sobel filter) using the smoothing effect by approximating a vertical/horizontal gradient. A binary image was finally obtained using the filtered image. Finally, total area of the connected region in the binary image, as well as local binary pattern (LBP) features were extracted (Table 2), which encode local texture information. LBP captures the grayscale relationships between each pixel and its surrounding neighborhood pixels in the image, encoding this local information into a binary number to characterize local texture patterns [9]. LBP histogram containing 10 bins was finally obtained in this study.

### Modelling and evaluation

#### Machine learning models.

Linear and non-linear classifiers were both employed in this study. In total, four traditional classifiers were evaluated in this study, namely k-nearest neighbors (kNN), multilayer perceptron (MLP), linear discriminant analysis (LDA), and logistic regression (LR).

The kNN classifier finds a group of *k* objects in the training set that are closest to the test object. Given a training set *D* and a test object ***x***=(*x*_test_, *y*_test_), kNN computes the distance between *z* and all the training objects (*x*_train_, *y*_train_) ∈ *D* to determine its nearest-neighbor list *D*_z_. Once the nearest-neighbor list is obtained, the test object is classified based on the majority class of its nearest neighbors [10]. In this study, *x*_train_ and *x*_test_ are the image features of base load image of a training and test object, respectively, while *y*_train_ and *y*_test_ are their corresponding classes. In this study, the distance weighting method was used to assign weights to the neighbors. MLP is a feedforward artificial neural network composed of multiple layers of simple, two-state, sigmoid processing elements that interact using weighted connections. The information propagates sequentially from the input layer forward to the output layer without backward connections. Three main components of the MLP include input layers, hidden layers, and output layers. The input layer receives the image features of base load images *x*_train_, the output layers are viewed as the *y*_train_, and the hidden layers are the classifying layers to produce output values from the inputs, where the connection weights are adjusted to optimize the best results with the least difference [11]. In this study, two hidden layers with 80 and 50 neurons were employed. The maximum number of iterations was set to 1000, and early stopping was used to avoid overfitting. The *lbfgs* optimization solver was employed. LDA is a subspace technique that optimizes the Fisher score. It finds a linear transformation matrix that reduces the dimensionality of the feature space, which is selected to fulfill a given maximization criterion of separability among class distributions. The Fisher criterion is based on maximizing the distance among the means of the classes while minimizing their interclass variances [12]. In this study, the default SVD solver from scikit‑learn was employed, with the tolerance set to its default value of 0.0001. LR was primarily used for binary classification task. It transforms the probability of the binary outcome, which ranges from 0 to 1, into a continuous variable using the logit function, allowing for linear modeling. The logit function is defined as the natural logarithm of the odds of the outcome, enabling the relationship to be approximated as a straight line. Parameters in logistic regression are typically estimated using maximum likelihood estimation, which maximizes the probability of observing the given data [13]. When addressing multi-class classification problems, LR iteratively performs binary classification and outputs probabilistic outcomes. In this study, the maximum number of iterations was set to 1000. The hyperparameter *C* was set to 1.0, and the *lbfgs* optimization solver was employed. All the base load age classification models were developed using Python 3.10.

### Dataset preparation and evaluation metrics

Prior to training the classification model, the raw dataset (containing 200 samples) was split into training and test sets. Specifically, the training set consisted of 80% (32 images) randomly selected from each cycle class, while holding out the remaining 20% of the data for the test set. To evaluate the stability of the model, 5-fold cross-validation was performed. The performance of the machine learning models was evaluated using the following metrics:


$$Accuracy\left(ACC\right)=\frac{TP+TN}{TP+TN+FP+FN}$$ (1)

$$Recall\left(R\right)=\frac{TP}{TP+FN}$$ (2)

$$Precision\left(P\right)=\frac{TP}{TP+FP}$$ (3)

$$F1Score=\frac{2\times TP}{2\times TP+FP+FN}$$ (4)

where *TP*, *TN*, *FP*, *FN* are true positive, true negative, false positive, and false negative, respectively.

## Results

### Image of the base load

**Fig 3:**
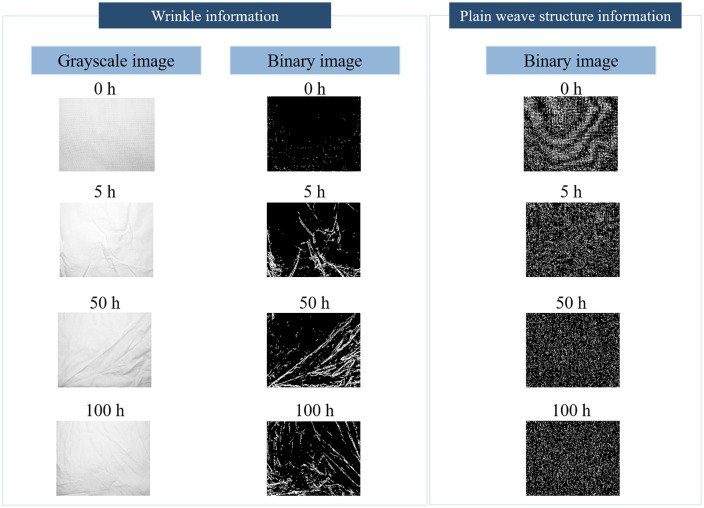
Examples of wrinkle information and plain weave structure information.

Fig. 3 shows the images of wrinkle information and plain weave information for the base load with ages of 0, 5, 50, and 100 hours. From the wrinkle information, it can be observed that after 5 h of operation, the grayscale image of the base load appears brighter compared to its unused state, while at 50 h and 100 h, the color becomes grayer. This phenomenon may occur because detergent has a bleaching effect on the fabric, and wrinkles at 5 h are significantly fewer than those at 50 h and 100 h, resulting in a smoother surface that reflects more light during imaging, thus appearing brighter. In the binary image representing wrinkle information of the base load, it can be observed that the number of wrinkles increases significantly as the load age extends from 0 h to 50 h. The number of wrinkles shows no significant difference between base loads for 50 h and 100 h, whereas the 100h sample appears to exhibit shorter, finer wrinkle segments. In the binary image extracted representing the plain weave structure information, it can be seen that the base load at 0 h age exhibits a well-defined warp-weft texture structure. After 5 h of operation, this textile architecture begins to alter. At 50 h age, pixels with value 1 (white) appear slightly reduced compared to the 5 h base load, while the 100 h base load sample shows negligible visual discrepancy from the 50 h state. The white and black pixels in the binary image can be viewed as the yarn and inter-yarn space of the base load, respectively. The reduction of the connected area (expressed by white pixels) should be related to the fiber breakage during the washing process in the washing machine.

### Grading of base load ages

Base load age grading models were developed using wrinkle information, plain weave structure information, and the combination of both, respectively, using kNN, MLP, LDA, and LR classifiers. The mean values and standard deviations of *ACC*, *R*, *P*, and *F*1*Score* obtained from five-fold cross-validation for the four classifiers are also summarized in Table 3. It can be observed that, regardless of the classifier used, the combined utilization of wrinkle information and plain weave structure information yields superior classification performance compared with using either type of information alone. Among the four classifiers, LR achieves the highest mean *ACC*, *R*, *P*, and *F*1*Score*, reaching 0.642, 0.642, 0.657, and 0.631, respectively. However, this model exhibits relatively large standard deviations, indicating greater performance variability. In addition, models constructed based on plain weave structure information outperform those based solely on wrinkle information.

**Table 3:** Validation results of the base load age grading models using different classifiers.

Classifier	Feature	*ACC*	*R*	*P*	*F*1*Score*
**kNN**	Wrinkle information	0.388 ± 0.052	0.388 ± 0.052	0.421 ± 0.081	0.382 ± 0.056
	Plain weave structure information	0.455 ± 0.107	0.455 ± 0.107	0.451 ± 0.096	0.442 ± 0.098
	Total information	0.539 ± 0.052	0.539 ± 0.052	0.521 ± 0.065	0.518 ± 0.049
**MLP**	Wrinkle information	0.418 ± 0.078	0.418 ± 0.078	0.430 ± 0.079	0.414 ± 0.077
	Plain weave structure information	0.442 ± 0.106	0.442 ± 0.106	0.446 ± 0.121	0.431 ± 0.111
	Total information	0.576 ± 0.051	0.576 ± 0.051	0.572 ± 0.054	0.562 ± 0.051
**LDA**	Wrinkle information	0.491 ± 0.045	0.491 ± 0.045	0.519 ± 0.067	0.488 ± 0.058
	Plain weave structure information	0.503 ± 0.041	0.503 ± 0.041	0.506 ± 0.051	0.489 ± 0.046
	Total information	0.576 ± 0.054	0.576 ± 0.054	0.598 ± 0.070	0.573 ± 0.061
**LR**	Wrinkle information	0.442 ± 0.068	0.442 ± 0.068	0.431 ± 0.092	0.427 ± 0.082
	Plain weave structure information	0.503 ± 0.031	0.503 ± 0.031	0.481 ± 0.052	0.479 ± 0.040
	Total information	0.642 ± 0.062	0.642 ± 0.062	0.657 ± 0.062	0.631 ± 0.068

The *ACC*, *R*, *P*, and F1Score of the models using the test dataset are presented in Table 4. Models utilizing the sum of the wrinkle and plain weave structure information consistently outperform those using single-type information, regardless of classifier selection, which is consistent with the results obtained from cross-validation. In addition, the plain weave structure information demonstrates greater contribution to base load age grading than the wrinkle structure information, resulting in better information for all classifiers. In the LDA-based models, classification performance using solely plain weave structure data was nearly identical to that of models incorporating both feature types. Among the four types of classifiers, the LR achieved the best performance for grading base load age, resulting in an *ACC*, *R*, *P* and *F*1*Score* of 0.775, 0.775, 0.783, and 0.773, respectively.

**Table 4:** Test results of the base load age grading models using different classifiers.

Classifier	Feature	*ACC*	*R*	*P*	*F*1*Score*
**kNN**	Wrinkle information	0.375	0.375	0.373	0.367
	Plain weave structure information	0.425	0.425	0.494	0.441
	Total information	0.550	0.550	0.563	0.552
**MLP**	Wrinkle information	0.475	0.475	0.469	0.470
	Plain weave structure information	0.575	0.575	0.570	0.569
	Total information	0.675	0.675	0.670	0.669
**LDA**	Wrinkle information	0.400	0.400	0.397	0.377
	Plain weave structure information	0.475	0.475	0.530	0.479
	Total information	0.475	0.475	0.547	0.479
**LR**	Wrinkle information	0.450	0.450	0.451	0.447
	Plain weave structure information	0.650	0.650	0.711	0.663
	Total information	0.775	0.775	0.783	0.773

**Fig 4:**
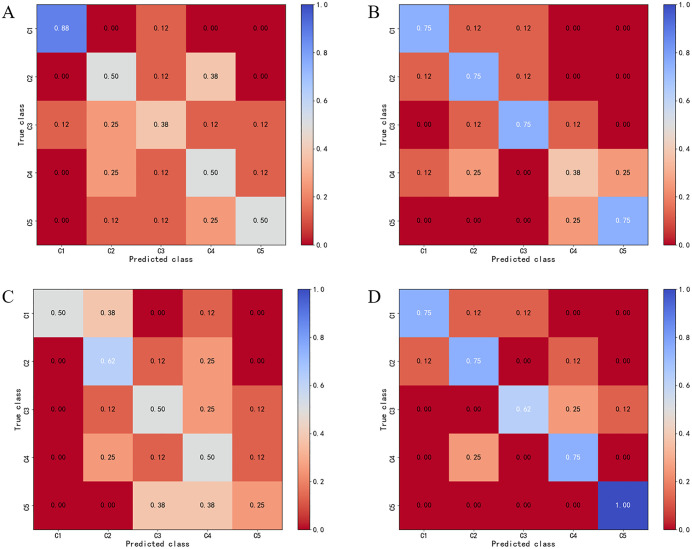
Confusion matrices for different classifier. (A) kNN. (B) MLP. (C) LDA. (D) LR.

The confusion matrices of the models utilizing the sum of the wrinkle and plain weave structure information in the test dataset are presented in Fig. 4. The running ages of 1–20 cycles, 21–40 cycles, 41–60 cycles, 61–80 cycles, and 81–100 cycles, were expressed as C1, C2, C3, C4, and C5, respectively. LR achieved high classification accuracy across all classes and performed especially well in the C5 category, with accuracies of 0.75, 0.75, 0.62, 0.75, and 1.00 for C1, C2, C3, C4, and C5, respectively. Two non-linear classifiers (as can be seen in Fig. 4(A) and 4(B)) produced similar performance in the C1 category. MLP classifiers achieved a classification accuracy of 0.75 across most classes except for C4.

The training and testing time for all classification models are presented in Table 5. It can be observed that the MLP has the longest training time, followed by LR, while both kNN and LDA have very short training times, approximately 1 ms. In terms of prediction time, MLP, LDA, and LR all exhibit very short prediction times, whereas kNN requires 62.03 ms for prediction. This is likely because kNN only stores the training set during the training phase without building a model, but during the prediction phase, it needs to perform distance calculations and select the top *k* smallest values, essentially executing a nearest neighbor search on the stored training set.

**Table 5:** Computational time for all classification models.

Parameters	kNN	MLP	LDA	LR
**Training time/ms**	1.03	57.03	1.00	11.28
**Testing time/ms**	62.03	0.00	0.00	1.01

## Discussion

**Fig 5:**
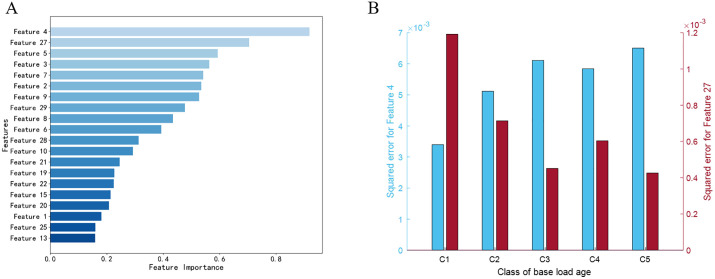
Illustration of feature importance in the LR model.

To illustrate feature importance in the LR model based on the combined wrinkle and plain-weave structure features, the importance scores were computed and the top 20 important features are presented in Fig. 5(A). The most important feature for base load age grading is the LBP feature (Feature 4, the corresponding name can be seen in Table 2) of the binary image of plain weave structure information. In addition, Feature 7, which is the mean color value of grayscale base load image, also makes a significant contribution to the grading of base load age. All features extracted from the binary image to characterize the plain weave structure of the base load fabric ranked among the top 20 most important, with most also ranking within the top ten. This appears to be the reason why models based on the plain weave structure information outperform those based on wrinkle information.

To further investigate the patterns of the top two significant features extracted from base loads of each category, the squared error between these features (mean value) and those of the 0h base load images was calculated, as can be seen in Fig. 5(B). It can be observed that with increasing base load age, the difference in the LBP feature (Feature 4) between the washed base load and the 0 h base load exhibits an overall increasing trend, while the difference in the mean color value of grayscale image between the washed base load and the 0h base load shows an overall decreasing trend. The C3 class disrupts the overall linear trend for both Feature 4 and Feature 27, which likely explains why the LR classifier achieved the lowest recognition accuracy for Class C3.

(A) Top 20 important features for LR. (B) Squared error of feature 4 (marked in blue) and feature 27 (marked in red) between 0h and 1–20 cycles base load (C1), 0h and 21–40 cycles base load (C2), 0h and 41–60 cycles base load (C3), 0 h and 61–80 cycles base load (C4), 0 h and 81–100 cycles base load (C5).

**Fig 6:**
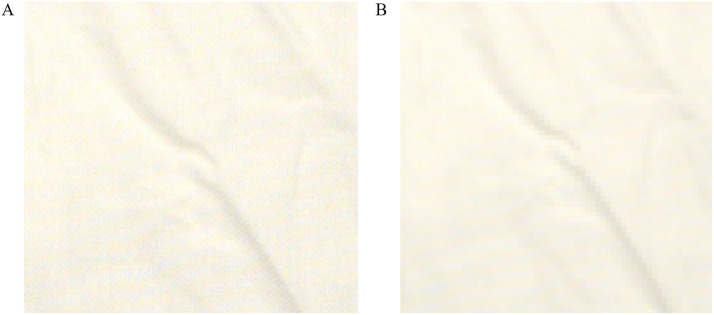
Locally magnified view of the base load image. (A) Original base load image (1280 × 1024). (B) Image after first cropping it to 1024 × 1024 centered on the image origin and then scaling it down to 224 × 224.

Compared with the complex feature extraction of traditional machine learning methods, the deep learning models use convolutional neural networks to extract the image features by self-learning. Many deep networks have been proven to exhibit better classification or prediction performance than traditional methods [14,15]. However, for the task of classifying the base load age, the accuracy of classification depends on both the macroscopic wrinkle skeleton and the microscopic plain weave structures (as shown in Table 4). The fixed-size input required by deep networks may significantly affect the classification performance of deep learning models. For currently popular deep learning networks, images are typically required to be input at a size of 224 × 224. Fig 6 shows the locally magnified view of the original base load image (1280 × 1024) and image after first cropping it to 1024 × 1024 centered on the image origin and then scaling it down to 224 × 224. As can be seen, the plain weave structures visible in Fig 6(A) are almost entirely absent in Fig 6(B), which greatly limits the information that deep networks can learn. Therefore, the base load age prediction results of deep networks are likely to be inferior to those of the LR model based on image features proposed in this study. S2 and S3 Figs present the training loss curves and confusion matrices obtained using ResNet50 and EfficientNetB1, respectively. The overall classification accuracies of ResNet50 and EfficientNetB1 are 0.626 and 0.400, respectively. For ResNet50, the classification accuracies for the five age grades (C1–C5) are 1.00, 0.38, 0.25, 0.75, and 0.75, respectively. For EfficientNetB1, the corresponding accuracies for C1–C5 are 0.88, 0.50, 0.12, 0.25, and 0.25, respectively. To ensure that the input image size matched the network requirement of 224 × 224, the original 1280 × 1024 images were first center-cropped to 1024 × 1024 and subsequently resized to 224 × 224. For both networks, the batch size was set to 16, the optimizer was Adam, and the learning rate was 0.001.

Model validation conducted on the current dataset suggests that, with appropriate feature extraction methods and classifiers, base load age can be effectively classified into different levels. Specifically, the results from all four classifiers may indicate that plain weave structure information contributes more effectively to the age grading than wrinkle information, and the combined use of both yields superior prediction performance. This consistent, non-accidental outcome confirms the rationality of the extracted features. Furthermore, all four models have been validated on an independent test set, suggesting that the LR model exhibits a degree of reliability. For subsequent applications, priority should be given to expanding the dataset. While this expansion may influence classification accuracy to some extent, the selected features and models are expected to remain applicable based on the current findings.

## Conclusion

This study investigated the application of computer vision techniques for grading the age of cotton base loads used in washing machine performance testing. A computer vision system was developed to capture images of base loads subjected to varying washing cycles (1–20, 21–40, 41–60, 61–80, and 81–100 cycles). Wrinkle information and plain weave structure information were extracted from the raw base load images. Color, texture, and area features were obtained to characterize the two types of information and used to train machine learning classifiers. Four types of classifiers including kNN, MLP, LDA, and LR, were compared. LR achieved high classification accuracy across all classes and performed especially well in the C5 category, with accuracies of 0.75, 0.75, 0.62, 0.75, and 1.00 for C1 (1–20 cycles), C2 (21–40 cycles), C3 (41–60 cycles), C4 (61–80 cycles), and C5 (81–100 cycles), respectively. Regardless of the classifier type, models employing exclusively plain weave structure information-derived features achieved superior performance compared to models employing exclusively wrinkle information-derived features. Additionally, it was discovered that the LBP feature extracted from the binary image of plain weave structure information is the most important feature in the LR model. The difference in the LBP feature between the washed base load and the new base load exhibited an overall increasing trend with extended washing time. The proposed approach could streamline the management of washing machine performance testing and contribute to more sustainable standard testing processes. Future study could be conducted on expanding the sample size and exploring the feasibility of using a mobile phone for measurements.

## Supporting information

S1 Table Image features. (XLSX)

S1 Fig Illustration of alternating orientation. (TIF)

S2 Fig Loss and accuracy of (A) ResNet50 and (B) EfficientNetB1. (TIF)

S3 Fig Confusion matrix of (A) ResNet50 and (B) EfficientNetB1. (TIF)
