# Supplementary figures and images for: A computer vision approach for the grading of cotton base load ages in measuring the performance of washing machine
Source: PLoS One. 2026 Apr 10;21(4):e0342045. doi: 10.1371/journal.pone.0342045 (PMC13068324; doi:10.1371/journal.pone.0342045)

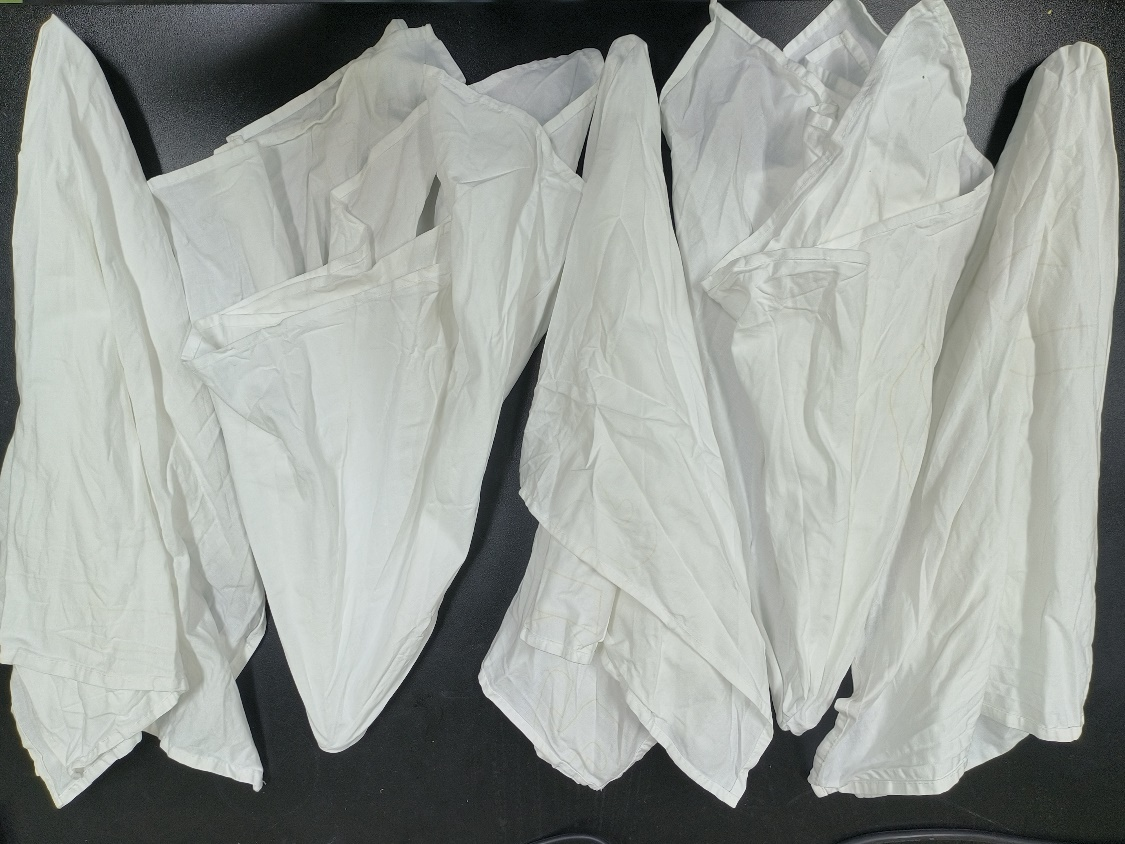

Supplement: S1 Fig — (TIF) [file pone.0342045.s002.tif]

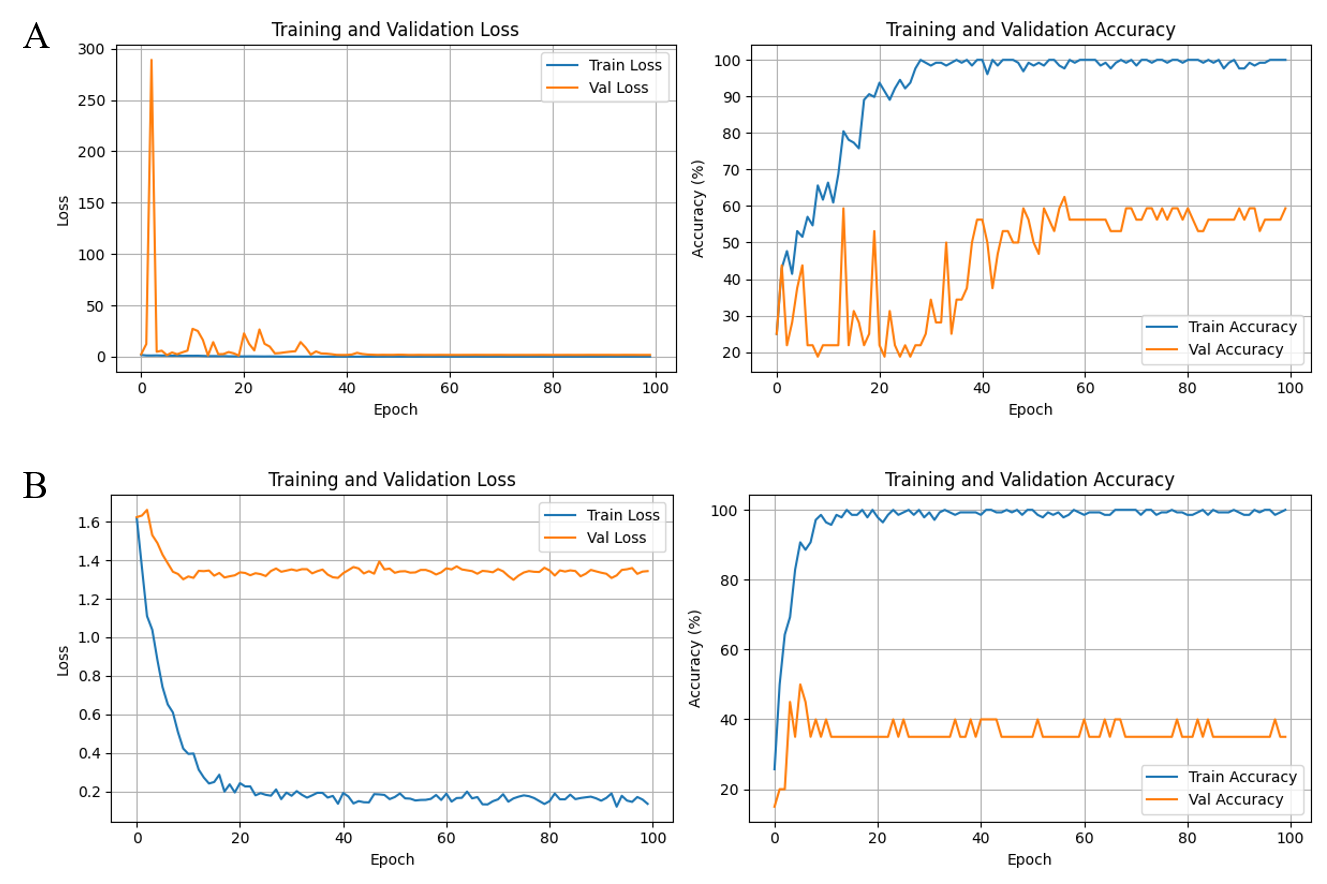

Supplement: S2 Fig — (TIF) [file pone.0342045.s003.tif]

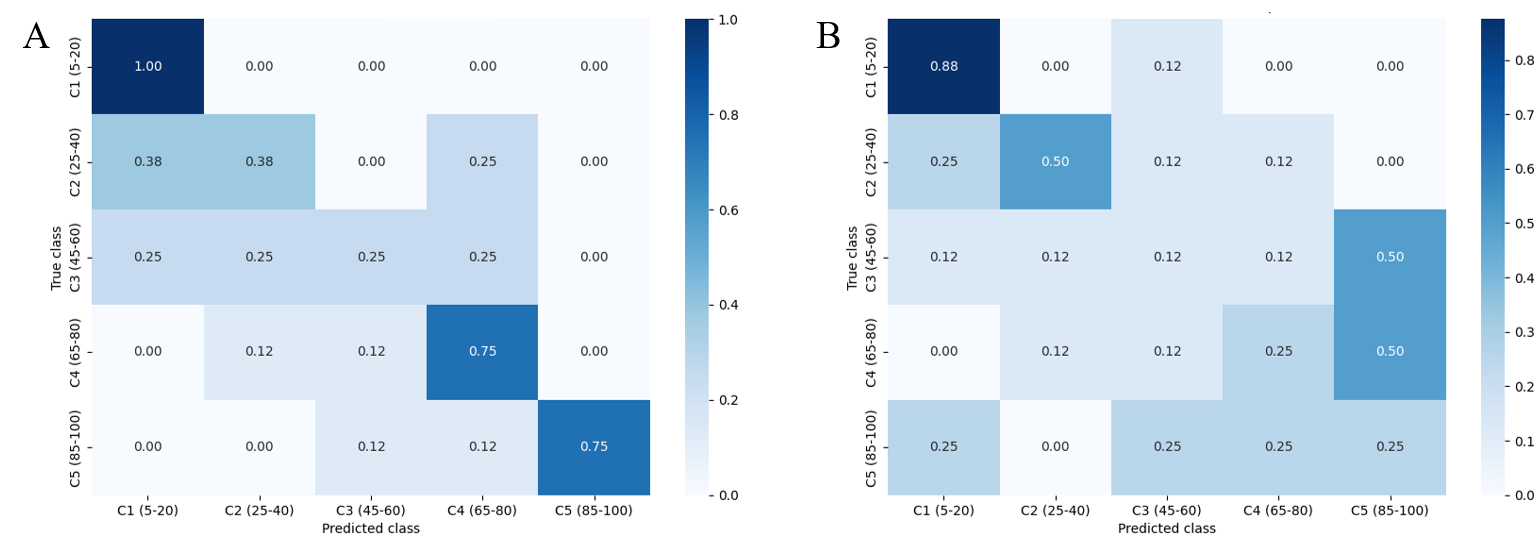

Supplement: S3 Fig — (TIF) [file pone.0342045.s004.tif]
